# UNDERSTANDING THE IMPACT OF CARE LITERACY ON PREVENTATIVE CARE: EVIDENCE FROM FAMILY CARERS IN JAPAN

**DOI:** 10.1093/geroni/igac059.2102

**Published:** 2022-12-20

**Authors:** Hiroko Costantini

**Affiliations:** University of Tokyo, Tokyo, Tokyo, Japan

## Abstract

To address the importance of family carers’ understanding of care, encompassing their own care as well as the broader care and health social systems, this paper leverages the notion of ‘care literacy’. The aim of this study is to understand the variation in care literacy and the impact of care literacy on preventative care. The empirical focus is on working family carers for older relatives in Japan, through a cross-sectional online survey that includes a novel operationalization of care literacy, established measure of health literacy, assessment of information used to understand care, and measures of preventative care. The participants’ (n = 292) mean age was 53, with 44% women, and an average of 8.3 hours per week caring for their parent(s). The measure of care literacy is shown to be correlated, as expected, but distinct to health literacy (correlation 0.60). Based on regression analysis of care literacy, significant explanatory variables are health literacy (p< 0.001), gender (p=0.044), number of sources of information on care (p=0.029), and care hours (p< 0.001). In contrast, proximity in living arrangements of carer and care receiver, and severity of care needs were not significant predictors. Turning to the impact of care literacy, care literacy is a significant explanatory variable for use of preventative measures (p=0.002), in particular related to nutrition (p< 0.001), frailty (p=0.028), dementia (p=0.090) and general home renovations (p=0.018). The pattern of results from this cross-sectional analysis indicates the importance of understanding the potential for improved care literacy as an enabler of better care.

